# Risk of Venous Thromboembolism in Patients with Presumed Primary Raynaud's Syndrome: A Propensity-Matched Study Using the TriNetX Database

**DOI:** 10.7150/ijms.139691

**Published:** 2026-09-03

**Authors:** Shang-Hsuan Huang, Chao-Bin Yeh, Yu-Hsun Wang, Shun-Fa Yang, Ying-Cheng Chen

**Affiliations:** 1 Department of Emergency Medicine, Taichung Armed Forces General Hospital, Taichung, Taiwan.; 2 School of Medicine, National Defense Medical University, Taipei, Taiwan.; 3 Institute of Medicine, Chung Shan Medical University, Taichung, Taiwan.; 4 Department of Emergency Medicine, School of Medicine, Chung Shan Medical University, Taichung, Taiwan.; 5 Department of Emergency Medicine, Chung Shan Medical University Hospital, Taichung, Taiwan.; 6 Department of Medical Research, Chung Shan Medical University Hospital, Taichung, Taiwan.; 7 Department of Surgery, Changhua Christian Hospital, Changhua, Taiwan.; 8 Department of Post-Baccalaureate Medicine, College of Medicine, National Chung Hsing University, Taichung, Taiwan.

**Keywords:** Raynaud's syndrome, venous thromboembolism, deep vein thrombosis, pulmonary embolism, TriNetX

## Abstract

**Background:**

Raynaud's syndrome (RS) is traditionally considered a benign vasospastic disorder; however, emerging evidence suggests that it may reflect systemic vascular dysfunction and a prothrombotic state. Emerging epidemiological evidence suggests an association between RP and VTE, but further validation across different populations and analytical approaches is needed.

**Methods:**

We conducted a retrospective cohort study with the TriNetX global electronic health record database. Adult patients diagnosed with RS between 2015 and 2024 were identified and matched 1:1 with individuals without RS by using propensity score matching, adjusting for demographics, comorbidities, medical utilization, and laboratory parameters. Patients with autoimmune diseases, malignancy, thrombophilia, vascular disease, pregnancy, or anticoagulant and hormonal contraceptive use were excluded to minimize confounding. The primary outcome was incident VTE, including deep vein thrombosis (DVT) and pulmonary embolism (PE), assessed with Cox proportional hazards models.

**Results:**

After matching, 93,263 patients were included in each group. During follow-up, VTE occurred in 1,407 patients in the RS group and 1,179 patients in the non-RS group. RS was associated with a significantly increased risk of VTE (hazard ratio [HR] 1.33; 95% confidence interval [CI] 1.24-1.44). Increased risks were observed for both DVT (HR: 1.30; 95% CI: 1.18-1.43) and PE (HR: 1.38; 95% CI: 1.24-1.54). Subgroup analyses showed a more pronounced association in younger individuals and consistent effects across sexes.

**Conclusions:**

RS is associated with an increased risk of venous thromboembolism independent of major confounding conditions.

## Introduction

Raynaud's syndrome (RS), named after Maurice Raynaud, first described in 1862 1, is a vasospastic disorder characterized by episodic digital ischemia precipitated by cold exposure or emotional stress. The estimated prevalence of RS ranges from 3% to 5% in the general population and is substantially higher among younger adults and women, particularly in colder climates 2, 3. RS is classified into primary and secondary types. Primary RS occurs in the absence of identifiable systemic disease, whereas secondary RS is usually associated with systemic diseases, particularly connective tissue diseases 4-7.

Traditionally, RS has been considered a functional peripheral vascular disease, but increasing evidence suggests that it reflects a broader systemic vascular abnormality, rather than a localized phenomenon. Previous pathophysiological studies have found that vasospasm is associated with endothelial dysfunction, decreased nitric oxide bioavailability, increased sympathetic vasoconstrictive activity, and microvascular structural changes 8-10. These mechanisms continue to form the basis of contemporary models of RS pathophysiology 7. In addition to vasospasm, RS patients exhibit hematological changes, including coagulation abnormalities such as increased blood viscosity, elevated fibrinogen levels, and enhanced platelet activation 11-15. More recent work has emphasized the importance of fibrin clot structure and impaired fibrinolysis in thrombotic disease, highlighting the role of dense fibrin networks and reduced clot permeability in determining thrombotic risk 16, 17. These studies suggest that RS may be related to a systemic environment prone to thrombosis.

Previous observational studies have found an association between RS and adverse cardiovascular events 18. Population cohort studies have reported that patients with RS have a higher incidence of cardiovascular disease and a higher long-term mortality rate, which supports the hypothesis that RS may be a potential marker of vascular disease 19, 20. A systematic review by Garner *et al*. also highlighted the association between primary RS and cardiovascular risk factors, but did not systematically assess whether venous thromboembolism (VTE) was involved 21. VTE, comprising deep vein thrombosis (DVT) and pulmonary embolism (PE), is a multifactorial thrombotic disorder influenced by both acquired and inherited risk factors and remains an important cause of cardiovascular morbidity and mortality 22.

The study by Żuk *et al*. also provided more direct evidence linking RS to venous thrombosis. Patients with primary RS have adversely altered plasma fibrin clot properties, including decreased clot permeability and prolonged clot lysis time, both of which indicate impaired fibrinolytic function. Elevated levels of fibrinogen and von Willebrand factor also suggest an endothelial cell activation and prothrombotic fibrin clot phenotype. More importantly, RS can independently predict prolonged clot lysis time, supporting its role as a risk factor for VTE, particularly in women 23.

Recent epidemiological evidence has begun to support an association between RP and thromboembolic outcomes. Hughes *et al*. reported an increased risk of VTE among individuals with RP without recognized systemic autoimmune rheumatic diseases, with the association observed across younger and older age groups 24. However, further evidence is needed to characterize this relationship using alternative comparator populations, broader exclusion of established thrombotic conditions, and more detailed evaluation of VTE-specific outcomes and clinically relevant subgroups. Therefore, we conducted a large propensity score-matched cohort study to evaluate the association between presumed primary RP and incident VTE, including DVT and PE, while accounting for demographic characteristics, comorbidities, healthcare utilization, and laboratory parameters.

## Materials and Methods

### Data collection

This retrospective cohort study was conducted using the TriNetX Database, a large U.S.-based electronic health record network comprising more than 275 million individuals. The database contains de-identified, patient-level data from participating healthcare organizations, including demographic characteristics, diagnoses coded using the International Classification of Diseases, Tenth Revision, Clinical Modification (ICD-10-CM), laboratory results coded using Logical Observation Identifiers Names and Codes (LOINC), and medication records standardized using RxNorm and Anatomical Therapeutic Chemical (ATC) classification codes. The selection of laboratory parameters in this study was informed by prior research demonstrating impaired fibrinolysis, increased fibrin density, and endothelial activation in patients with primary Raynaud's syndrome, including elevated levels of fibrinogen, D-dimer, C-reactive protein, and von Willebrand factor.

This study involved a secondary analysis of pre-existing, de-identified data and was therefore exempt from informed consent requirements. The de-identification process complied with the standards outlined in Section §164.514(a) of the Health Insurance Portability and Accountability Act (HIPAA) Privacy Rule and was validated by a qualified expert in accordance with Section §164.514(b) 25. The study protocol was reviewed and approved by the Institutional Review Board of Chung Shan Medical University Hospital (IRB number: CS2-23180).

### Study participants and main outcome

The inclusion criteria for this study were adults aged ≥ 18 years between January 1, 2015, and December 31, 2024. The RS cohort consisted of patients diagnosed with RS (ICD-10-CM: I73.0). The index date was defined as the date of initial diagnosis of RS. The comparison cohort consisted of individuals who underwent general adult medical examination (ICD-10-CM: Z00.0) during the same period and had never been diagnosed with RS. The index date was defined as the date of the first general adult medical examination. Because ICD-10-CM code I73.0 does not reliably distinguish primary from secondary RS, presumed primary RS was operationally defined as RS without a documented secondary cause on or before the index date. Diagnoses occurring after the index date were not used to determine cohort eligibility, thereby avoiding the use of future information in exposure classification. Individuals with a prior diagnosis of systemic autoimmune diseases, malignancies, hereditary thrombophilia, or vascular diseases were excluded. In addition, patients who were pregnant or had exposure to hormonal contraceptives or coagulation-related medications on or before the index date were excluded to minimize potential confounding effects (Supplementary Table s1). Participants were excluded if they had any of the following diagnoses or treatments on or before the index date: a history of VTE (deep vein thrombosis or pulmonary embolism).

The primary outcome was new-onset VTE, defined as deep vein thrombosis of the lower extremities (ICD-10-CM: I82.4) and/or pulmonary embolism (ICD-10-CM: I26). Secondary outcome measures were analyzed separately for DVT and PE.

This study used a 1:1 propensity score matching (PSM) to balance baseline characteristics between the RS and non-RS groups. The matching process was conducted using the “Balance Cohorts” function in TriNetX, which employs 1:1 greedy nearest-neighbor matching with a caliper of 0.1 pooled standard deviations. Matching variables included: age, sex, race, body mass index (BMI), healthcare usage patterns (outpatient, emergency, inpatient), comorbidities (hypertension, hyperlipidemia, nicotine dependence, type 2 diabetes mellitus, chronic obstructive pulmonary disease, alcohol-related diseases), and baseline laboratory parameters related to coagulation and inflammation (e.g., D-dimer, fibrinogen, platelet count, C-reactive protein, prothrombin time (PT), international normalized ratio (INR), activated partial thromboplastin time (aPTT)) (Supplementary Table s2). All covariates were assessed using information available during the 1-year period preceding the index date.

### Statistical analysis

Baseline characteristics between the Raynaud's syndrome and non-Raynaud's syndrome groups were compared using standardized mean differences (SMDs), with an SMD < 0.1 indicating adequate covariate balance. Continuous variables were summarized as means with standard deviations (SDs), while categorical variables were presented as frequencies and percentages. The risk of venous thromboembolism was assessed using Kaplan-Meier survival curves, with between-group comparisons performed using Cox proportional hazards regression models to estimate hazard ratios and corresponding 95% confidence intervals. Stratified analyses were further conducted according to age, sex, and body mass index (BMI) to examine potential differences in venous thromboembolism risk across subgroups. Additional analyses were conducted to assess the robustness of the findings. First, the study population was stratified by calendar period into the pre-COVID-19 period (2015-2019) and the COVID-19 period (2020-2024) to examine whether the association between RS and VTE differed across these periods. Second, negative-control outcome analyses were performed using cataract (ICD-10-CM: H25), burns and corrosions of external body surface, specified by site (ICD-10-CM: T20-T25), and exposure to smoke, fire, and flames (ICD-10-CM: X00-X08) as outcomes. All statistical analyses were conducted using the TriNetX platform, which employs R software (version 4.0.2) as its underlying analytical engine.

## Results

### Baseline characteristics

Following inclusion and exclusion criteria, a total of 93,263 patients with RS and 6,970,456 individuals without RS were identified. After 1:1 propensity score matching, 93,263 pairs were included in the analysis (Figure 1). Baseline demographic characteristics, comorbidities, healthcare utilization, and laboratory findings were well balanced after matching, with SMD < 0.1. Table 1 shows the baseline demographic characteristics, comorbidities, healthcare use patterns, and laboratory findings of patients with and without RS. Compared to patients without RS, patients with RS were older, predominantly female, and had a lower body mass index (BMI). Furthermore, there were significant differences between the two groups in terms of racial distribution, healthcare use patterns, comorbidities, and several coagulation and inflammation-related laboratory findings, with SMD > 0.1 for multiple variables.

After 1:1 propensity score matching, 93,263 well-balanced paired samples were ultimately included for analysis. The two groups of patients were well-balanced in baseline demographic characteristics, comorbidities, medical use, and laboratory indicators. Most covariates achieved an SMD < 0.1; however, residual imbalance remained for several sparsely measured laboratory variables. These matching results also demonstrate the comparability of the paired cohorts at baseline, allowing subsequent analyses to assess the association more reliably between RS and the risk of VTE.

### Risk of venous thromboembolism

Table 2 presents the risk of VTE in patients with RS compared to the matched control group. During the follow-up period, 1407 patients in the RS group and 1179 patients in the non-RS group developed VTE. The risk of VTE was significantly higher in patients with RS than in those without (Hazard ratio [HR] 1.33; 95% Confidence interval [CI] 1.24-1.44). When analyzed separately, RS was associated with an increased risk of DVT (HR 1.30; 95% CI 1.18-1.43) and PE (HR 1.38; 95% CI 1.24-1.54). These results were consistent across all outcome measures, demonstrating a significant association between RS and the risk of VTE. As shown in Figure 2, the Kaplan-Meier analysis also revealed early and sustained differences in the cumulative incidence of VTE between the groups. In the stratified analysis by calendar period, the association between Raynaud's syndrome and venous thromboembolism (VTE) remained statistically significant across both the pre-COVID-19 (2015-2019) and 2020-2024 periods, with the former group consistently exhibiting a higher risk of VTE (Supplementary Table s3). In the negative-control outcome analyses, no statistically significant association was observed between Raynaud's syndrome and any of the prespecified negative-control outcomes, including cataract, burns and corrosions of the external body surface (specified by site), and exposure to smoke, fire, and flames (Supplementary Table s4).

### Subgroup analyses

Subgroup analyses of venous thromboembolism risk are shown in Figure 3. The increased risk associated with Raynaud's syndrome was generally consistent across predefined subgroups. A stronger association was observed among patients aged 18-64 years (HR 1.44; 95% CI 1.30-1.58) compared with those aged 65 years or older (HR 1.18; 95% CI 1.04-1.34). Increased risks were identified in both women (HR 1.32; 95% CI 1.20-1.44) and men (HR 1.29; 95% CI 1.09-1.52). When analyzed by race, higher hazard ratios were recorded for Asian individuals (HR 2.57; 95% CI 1.11-5.91) and Black or African American individuals (HR 1.58; 95% CI 1.19-2.09), though some subgroups had limited event numbers. When stratified by body mass index (BMI), the increased risk of VTE associated with RS was also observed among patients with normal weight and overweight status.

## Discussion

This propensity score-matched cohort study found that RS is independently associated with a higher risk of VTE, encompassing both DVT and PE. The results build on earlier mechanistic and observational evidence, offering population-level validation that RS is associated with a significant VTE risk.

The associations found in the study are biologically plausible and supported by a growing body of literature describing coagulation and endothelial abnormalities in patients with RS. Previous studies have shown that RS patients exhibit enhanced platelet activation, elevated fibrinogen concentrations, and altered hemorheology properties, suggesting a shift toward a prothrombotic state 11-14. Furthermore, endothelial dysfunction, marked by reduced vasodilatory responses and elevated levels of circulating markers indicative of endothelial damage, has been frequently observed in RS 8, 10, 26.

The results of our study align with the foundational research by Żuk *et al*., which showed that patients with primary RS form denser fibrin clots with reduced permeability and prolonged clot lysis time. These findings highlight impaired fibrinolysis and a prothrombotic fibrin clot profile. Moreover, the elevated von Willebrand factor levels reported in their study suggest a significant role for endothelial activation or injury as a key mechanism linking RS to thrombosis. Importantly, RS was identified as an independent predictor of prolonged clot lysis time, even after adjusting for fibrinogen, indicating an inherent prothrombotic effect that surpasses traditional risk factors 23. Longitudinal studies have previously indicated that RS could represent an early clinical sign of systemic vascular dysfunction, potentially appearing years before the onset of overt cardiovascular or thrombotic conditions 19. Recent studies have also found that pediatric patients with primary Raynaud's phenomenon had an increased risk of atherosclerosis compared with healthy children 27. From this perspective, RS may serve as a clear and identifiable clinical indicator of increased thrombotic risk.

The study by Żuk *et al*. evaluated fibrin clot properties following a VTE event. Building on this, our research extends these insights by showing that RS not only precedes but also predicts the occurrence of VTE in a large real-world cohort. To reduce potential confounding factors from secondary causes of RS and related prothrombotic conditions, we excluded individuals with autoimmune diseases, malignancies, hereditary thrombophilia, pregnancy, hormonal therapy, and anticoagulant use. This approach enhances the evidence that RS may serve as an independent marker for venous thrombotic risk. Our findings are also consistent with recently published epidemiological evidence. Hughes *et al*. reported that RP without recognized systemic autoimmune rheumatic disease was associated with an increased risk of VTE, with HR of 1.32 among individuals younger than 45 years and 1.20 among those aged 45 years or older 24. The similar effect observed in the two studies strengthen the evidence for an association between RS and VTE across different cohort definitions and comparator populations.

Our study differs from Hughes *et al*. in several aspects. VTE was the primary outcome, with DVT and PE evaluated separately, and broader baseline exclusions were applied for conditions associated with secondary RP or thrombotic risk. The propensity score model also incorporated healthcare utilization and available laboratory parameters about coagulation and inflammation in addition to demographic characteristics and comorbidities. In addition, we examined associations across age, sex, race, and BMI subgroups. These analyses provide complementary evidence that the observed association between RP and VTE is not restricted to a single comparator population or analytical approach.

Subgroup analyses provide additional insight. The stronger link noted among younger individuals and women is particularly significant, consistent with earlier findings that RS tends to be more common and clinically pronounced within these groups 2, 6, 10. The stronger relative association observed in younger patients may partly reflect the lower background incidence of VTE in younger populations; as baseline VTE risk rises substantially with age 28, the relative contribution associated with RP may become less pronounced among older adults. In contrast, attenuation of the association among individuals with obesity may reflect the dominant thrombotic risk conferred by obesity itself 29, 30. A prior study found that lower body weight was linked to a higher risk of developing RP 31. The absence of a statistically significant association in individuals with low body mass index likely reflects limited statistical power and wider confidence intervals rather than the absence of effect. In conclusion, our findings advocate for a significant shift in the clinical perspective of RS. Rather than viewing it as a benign peripheral vasospastic disorder, it should be recognized as a systemic vascular condition with possible thromboembolic risks. Further prospective studies are needed to determine whether individuals with RS could benefit from improved VTE risk assessment, more rigorous monitoring, or preventive interventions.

This study has several strengths. The use of a large multinational electronic health record database enabled inclusion of a diverse population and improved generalizability. The retrospective cohort design with longitudinal follow-up allowed assessment of incident VTE, and propensity score matching achieved a good balance between groups. In addition, the use of real-world clinical data enhances the relevance of the findings. Several limitations should be acknowledged. First, residual confounding cannot be excluded because of the observational nature of this study. Although propensity score matching was performed to balance measured baseline characteristics, PSM cannot eliminate residual confounding from unmeasured or imperfectly measured factors. Residual confounding related to smoking, obesity, hormonal factors, physical mobility, and socioeconomic status may remain. Therefore, the observed association between RS and VTE should not be interpreted as evidence of an independent causal relationship. Second, the identification of RS relied on ICD-10-CM coding, which does not reliably distinguish primary from secondary disease. Although we excluded patients with documented conditions associated with secondary RS on or before the index date, some patients initially classified as having presumed primary RS may have had an occult secondary disorder that was recognized only during subsequent follow-up. We intentionally did not use diagnoses occurring after the index date to retrospectively redefine exposure status, because doing so would introduce future information into cohort classification and potentially result in selection bias. Therefore, residual misclassification between primary and secondary RS cannot be completely excluded. Third, important clinical variables, including RS disease severity, lifestyle factors, physical activity or mobility, and medication adherence, were unavailable or incompletely captured in the database. Incomplete laboratory data may also have introduced selection bias, particularly because laboratory measurements were not available for all patients. Fourth, differences in surveillance intensity may have resulted in detection bias, whereby patients with RS may have had more frequent healthcare encounters and therefore greater opportunities for VTE detection. Although healthcare utilization was incorporated into the propensity score model and the comparator cohort were restricted to individuals with documented general adult medical examinations, residual surveillance bias cannot be completely excluded. Fifth, competing risks could not be explicitly accounted for because the TriNetX platform does not currently support competing-risk analyses. Death may act as a competing event for the occurrence of VTE, and its impact could therefore not be formally modeled in the present analysis. This limitation affects the detailed assessment and reporting of the number of patients remaining under observation during follow-up.

RS is associated with a significantly increased risk of venous thromboembolism in adults, independent of major confounding conditions. This indicates that RS might be an underestimated risk factor for VTE and warrants further prospective studies and validation. Clinicians should consider thromboembolic risk assessment when managing patients with RS, particularly in younger individuals and high-risk subgroups.

## Supplementary Material

Supplementary tables.

## Figures and Tables

**Figure 1:**
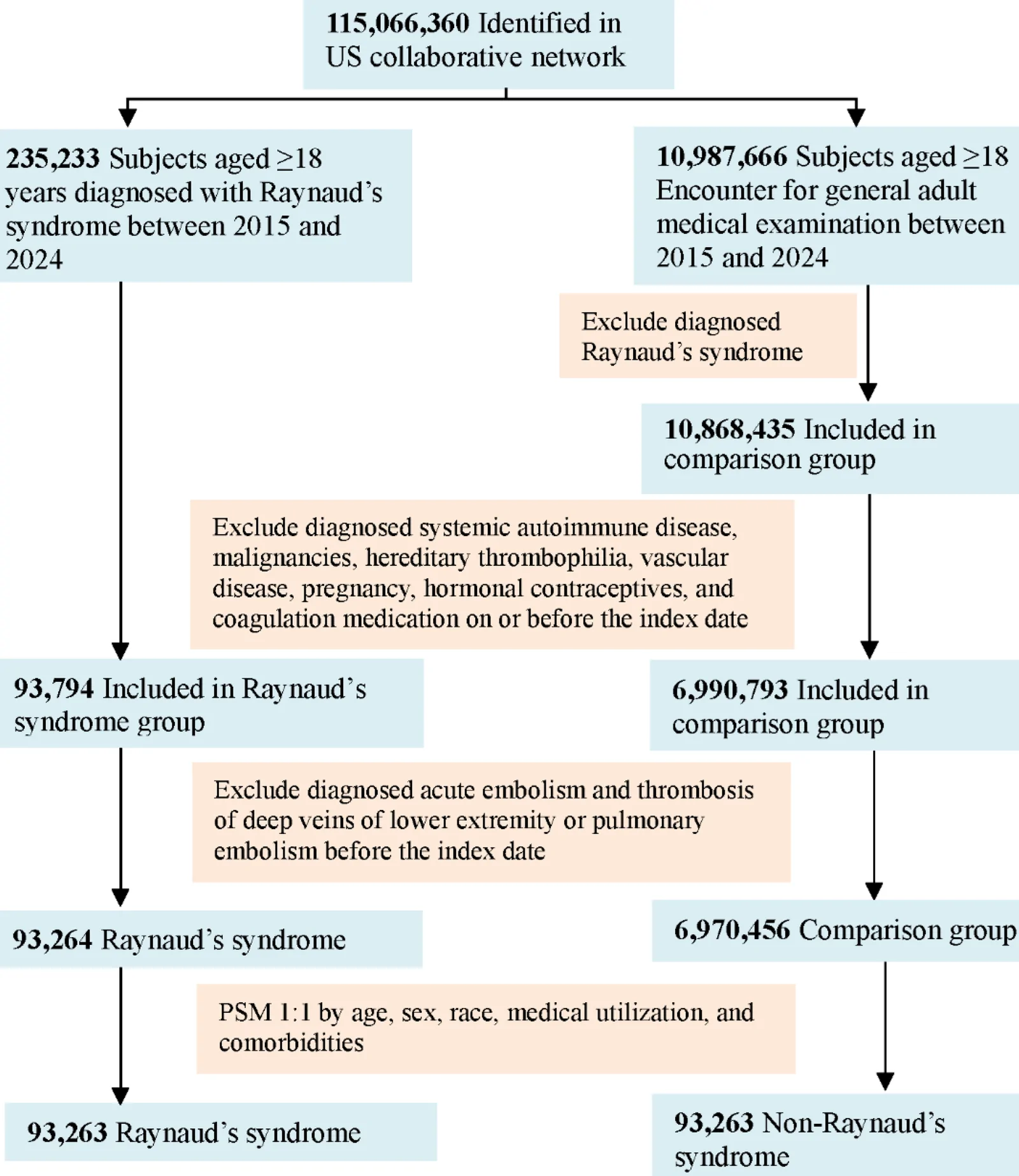
Flowchart illustrating participant selection

**Figure 2:**
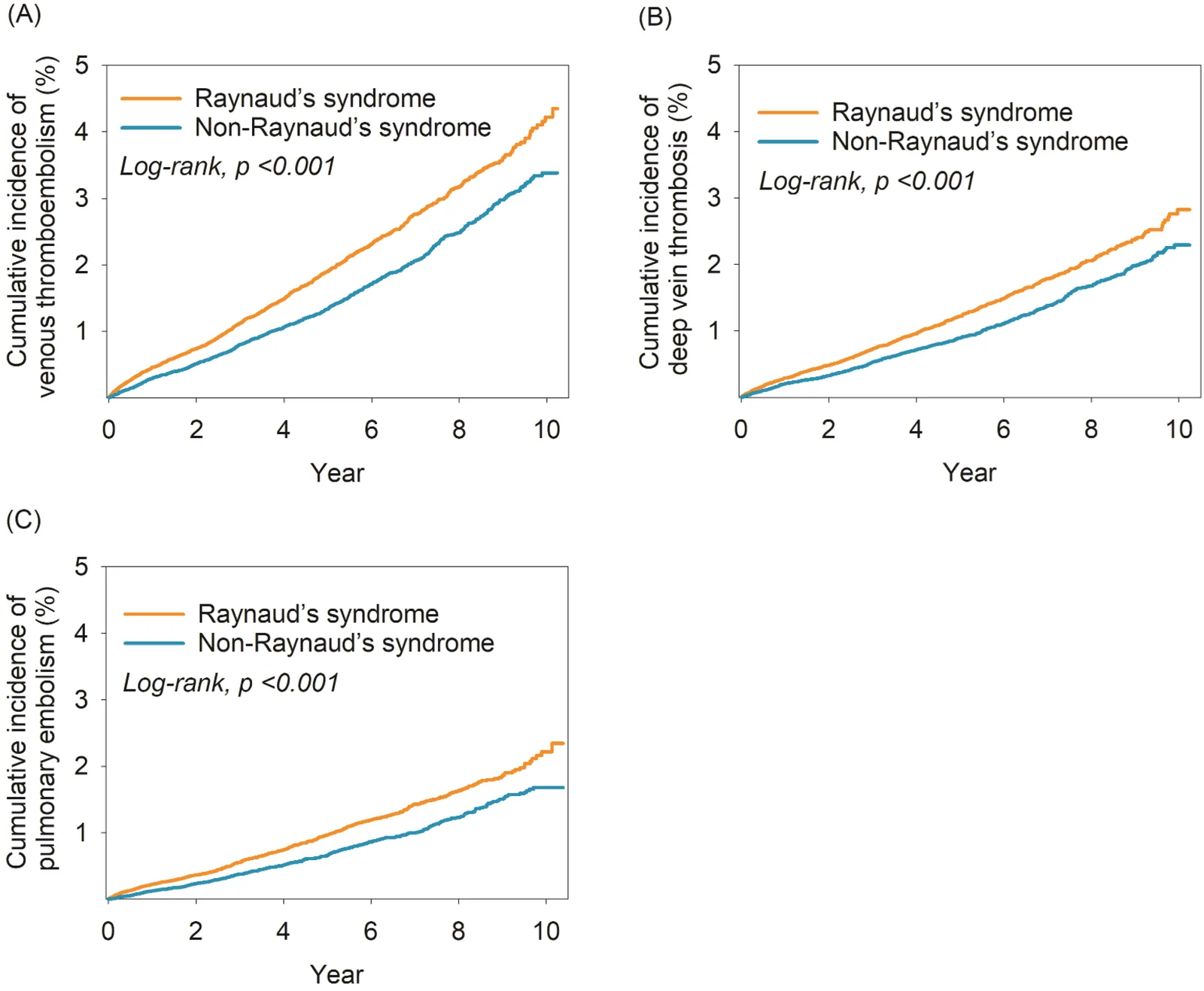
Kaplan-Meier curves showing the cumulative incidence of (A) venous thromboembolism, (B) deep vein thrombosis, and (C) pulmonary embolism in patients with and without Raynaud's syndrome.

**Figure 3:**
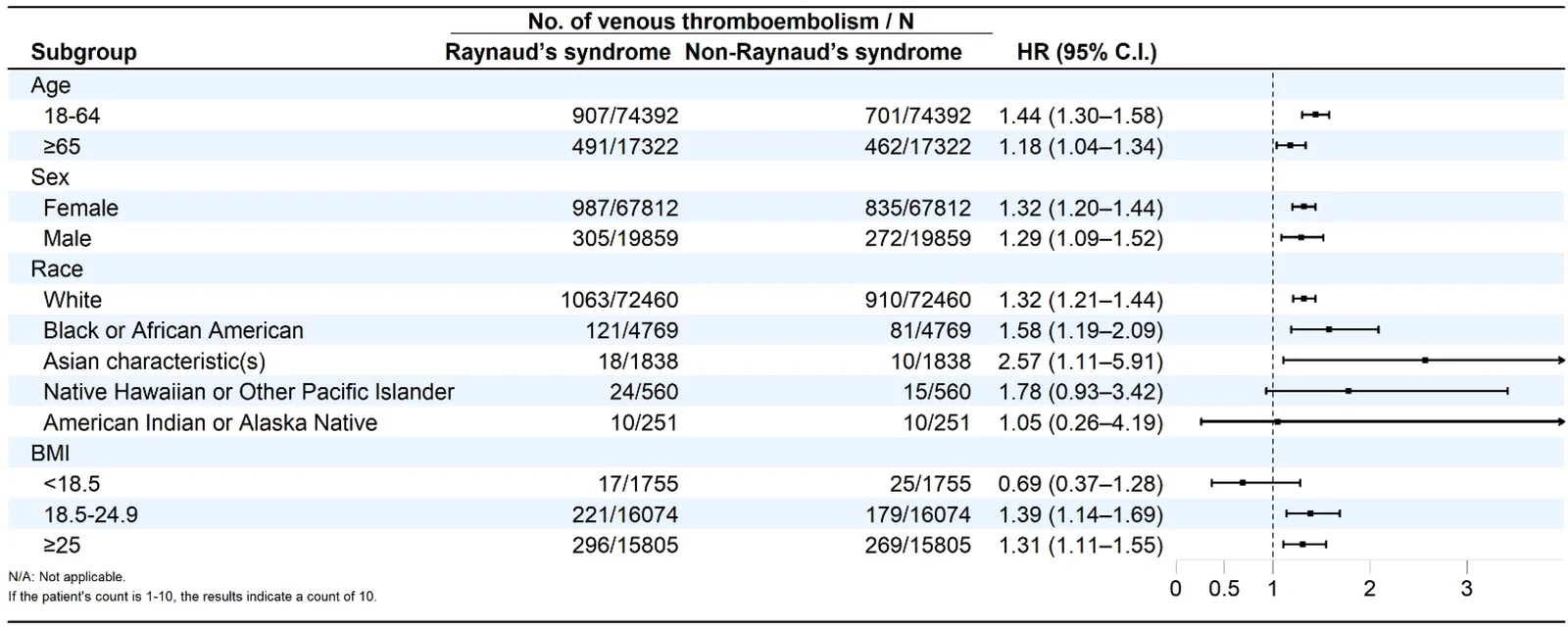
Forest plot comparing the risk of venous thromboembolism between Raynaud's syndrome and non-Raynaud's syndrome group.

**Table 1:** Demographic characteristics of Raynaud's syndrome group and non-Raynaud's syndrome group

	Before PSM			After PSM		
	Raynaud's syndrome N = 93264	Non-Raynaud's syndrome N = 6970456	SMD	Raynaud's syndrome N = 93263	Non-Raynaud's syndrome N = 93263	SMD
**Age, Mean ± SD**	48.26 ± 16.45	44.91 ± 17.07	0.200	48.26 ± 16.45	48.27 ± 16.45	0.001
**Sex**						
Female	67812 (72.71)	3327851 (47.74)	0.528	67811 (72.71)	67799 (72.70)	< 0.001
Male	19860 (21.29)	3171874 (45.51)	0.531	19860 (21.30)	21265 (22.80)	0.036
Unknown Gender	5592 (6.00)	470731 (6.75)	0.031	5592 (6.00)	4199 (4.50)	0.067
**Race**						
White	72460 (77.69)	4347581 (62.37)	0.339	72459 (77.69)	72489 (77.73)	0.001
Black or African American	4771 (5.12)	844080 (12.11)	0.251	4771 (5.12)	4760 (5.10)	0.001
Asian characteristic(s)	1841 (1.97)	370680 (5.32)	0.179	1841 (1.97)	1834 (1.97)	0.001
Native Hawaiian or Other Pacific Islander	560 (0.60)	45063 (0.65)	0.006	560 (0.60)	543 (0.58)	0.002
American Indian or Alaska Native	253 (0.27)	22591 (0.32)	0.010	253 (0.27)	258 (0.28)	0.001
Other Race	2232 (2.39)	275859 (3.96)	0.089	2232 (2.39)	2974 (3.19)	0.048
Unknown Race	11147 (11.95)	1064602 (15.27)	0.097	11147 (11.95)	10405 (11.16)	0.025
**BMI, Mean ± SD**	25.91 ± 6.21	29.27 ± 7.06	0.505	25.91 ± 6.21	26.30 ± 6.35	0.062
<18.5	17385 (18.64)	634186 (9.10)	0.279	17384 (18.64)	17405 (18.66)	0.001
18.5-24.9	10544 (11.31)	738446 (10.59)	0.023	10544 (11.31)	10544 (11.31)	< 0.001
≥25	17979 (19.28)	1597133 (22.91)	0.484	17979 (19.28)	17956 (19.25)	< 0.001
**Medical utilization**						
Ambulatory	58543 (62.77)	3683933 (52.85)	0.202	58542 (62.77)	58530 (62.76)	< 0.001
Emergency	6829 (7.32)	488093 (7.00)	0.012	6829 (7.32)	7131 (7.65)	0.012
Inpatient Encounter	4587 (4.92)	205804 (2.95)	0.101	4587 (4.92)	3360 (3.60)	0.065
**Comorbidities**						
Hypertension	10329 (11.08)	841257 (12.07)	0.031	10328 (11.07)	10972 (11.77)	0.022
Hyperlipidemia	5353 (5.74)	427996 (6.14)	0.017	5353 (5.74)	5941 (6.37)	0.026
Nicotine dependence	2372 (2.54)	158530 (2.27)	0.018	2372 (2.54)	2553 (2.74)	0.012
Type 2 diabetes mellitus	2010 (2.16)	300153 (4.31)	0.122	2010 (2.16)	1991 (2.14)	0.001
Chronic obstructive pulmonary disease	814 (0.87)	50272 (0.72)	0.017	814 (0.87)	933 (1.00)	0.013
Alcohol related disorders	644 (0.69)	43681 (0.63)	0.008	644 (0.69)	678 (0.73)	0.004
**Laboratory**						
Fibrin D-dimer DDU [Mass/volume]	25 (0.03)	957 (0.01)	0.113	25 (0.03)	23 (0.03)	0.063
Fibrinogen [Mass/volume]	113 (0.12)	2361 (0.03)	0.166	113 (0.12)	60 (0.06)	0.139
Platelets [#/volume] in Blood	3294 (3.53)	123382 (1.77)	0.054	3294 (3.53)	2400 (2.57)	0.026
von Willebrand factor (vWf) multimer	10 (0.01)	60 (0.00)	0.183	10 (0.01)	10 (0.01)	0.613
C reactive protein [Mass/volume]	4517 (4.84)	81037 (1.16)	0.154	4516 (4.84)	4507 (4.83)	0.110
INR in Blood	387 (0.42)	21788 (0.31)	0.236	387 (0.42)	375 (0.40)	0.191
Prothrombin time (PT)	1853 (1.99)	73753 (1.06)	0.141	1853 (1.99)	1226 (1.32)	0.075
Activated partial thromboplastin time (aPTT)	1097 (1.18)	36938 (0.53)	0.023	1097 (1.18)	641 (0.69)	0.021

PSM, propensity score matching; SD, standard deviation; SMD, standardized mean difference; BMI, body mass index; DDU, D-dimer Units; INR, international normalized ratio.

**Table 2:** Risk of venous thromboembolism exposed to Raynaud's syndrome group compared to non-Raynaud's syndrome group

	Raynaud's syndrome group		Non-Raynaud's syndrome group			P for proportional hazards assumption
	N	No. of event	N	No. of event	HR (95% CI)	
Venous Thromboembolism	93263	1407	93263	1179	1.33 (1.24-1.44)	0.002
Deep Vein Thrombosis	93263	914	93263	786	1.30 (1.18-1.43)	0.018
Pulmonary embolism	93263	716	93263	579	1.38 (1.24-1.54)	0.017

HR, Hazard Ratio; CI, Confidence interval.
