# Supplementary material for: Risk of Venous Thromboembolism in Patients with Presumed Primary Raynaud's Syndrome: A Propensity-Matched Study Using the TriNetX Database
Source: Int J Med Sci. 2026 Sep 3;23(10):3211–9. doi: 10.7150/ijms.139691 (PMC13617495; doi:10.7150/ijms.139691)
Supplement: Supplementary file 1 [file ijmsv23p3211s1.pdf]

Supplementary Table 1. Codes for exclusion criteria

Item	Code
Systemic autoimmune disease	
Systemic lupus erythematosus (SLE)	ICD10CM:M32
Other dermatomyositis, organ involvement unspecified	ICD10CM:M33.10
Polymyositis	ICD10CM:M33.2
Systemic sclerosis [scleroderma]	ICD10CM:M34
Other overlap syndromes	ICD10CM:M35.1
Systemic involvement of connective tissue, unspecified	ICD10CM:M35.9
Antiphospholipid syndrome	ICD10CM:D68.61
Malignancies	
Malignant neoplasms of lip, oral cavity and pharynx	ICD10CM:C00-C14
Malignant neoplasms of digestive organs	ICD10CM:C15-C26
Malignant neoplasms of respiratory and intrathoracic organs	ICD10CM:C30-C39
Malignant neoplasms of bone and articular cartilage	ICD10CM:C40-C41
Melanoma and other malignant neoplasms of skin	ICD10CM:C43-C44
Malignant neoplasms of mesothelial and soft tissue	ICD10CM:C45-C49
Malignant neoplasms of breast	ICD10CM:C50-C50
Malignant neoplasms of female genital organs	ICD10CM:C51-C58
Malignant neoplasms of male genital organs	ICD10CM:C60-C63
Malignant neoplasms of urinary tract	ICD10CM:C64-C68
Malignant neoplasms of eye, brain and other parts of central nervous system	ICD10CM:C69-C72
Malignant neoplasms of thyroid and other endocrine glands	ICD10CM:C73-C75
Malignant neoplasms of ill-defined, other secondary and unspecified sites	ICD10CM:C76-C80
Malignant neuroendocrine tumors	ICD10CM:C7A-C7A
Secondary neuroendocrine tumors	ICD10CM:C7B-C7B
Malignant neoplasms of lymphoid, hematopoietic and related tissue	ICD10CM:C81-C96
Hereditary thrombophilia	
Primary thrombophilia	ICD10CM:D68.5
Vascular	
Polyarteritis nodosa and related conditions	ICD10CM:M30
Other necrotizing vasculopathies	ICD10CM:M31
Vasculitis limited to skin, not elsewhere classified	ICD10CM:L95
Cerebral infarction	ICD10CM:I63
Occlusion and stenosis of precerebral arteries, not resulting in cerebral infarction	ICD10CM:I65
Occlusion and stenosis of cerebral arteries, not resulting in cerebral infarction	ICD10CM:I66
Ischemic heart diseases	ICD10CM:I20-I25
Atherosclerosis	ICD10CM:I70
Atheroembolism	ICD10CM:I75
Arterial embolism and thrombosis	ICD10CM:I74
Septic arterial embolism	ICD10CM:I76
Thromboangiitis obliterans [Buerger's disease]	ICD10CM:I73.1
Other specified peripheral vascular diseases	ICD10CM:I73.8
Peripheral vascular disease, unspecified	ICD10CM:I73.9
Pregnancy	
Pregnancy, childbirth and the puerperium	ICD10CM:O00-O9A
Under Hormonal therapies contraceptive use or oral hormone replacement therapy	
Hormonal contraceptives for systemic use	ATC: G03A
Coagulation medication	

Antithrombotic agents	ATC: B01A
Warfarin	RXNORM:11289
Dabigatran	RXNORM:1546356
Dabigatran etexilate	RXNORM:1037042
Tinzaparin	RXNORM:69646

ICD10CM: International Classification of Diseases, Tenth Revision, Clinical Modification.
ATC: Anatomical Therapeutic Chemical.
RxNorm: Medical prescription normalized Medical prescription.

Supplementary Table 2. Code of covariates

Item	Code
Comorbidities	
Hypertension	ICD10CM:I10
Hyperlipidemia	ICD10CM:E78.5
Nicotine dependence	ICD10CM:F17
Type 2 diabetes mellitus	ICD10CM:E11
Chronic obstructive pulmonary disease	ICD10CM:J44
Alcohol related disorders	ICD10CM:F10
Laboratory	
Fibrin D-dimer DDU [Mass/volume] in Platelet poor plasma	LOINC:48066-5
Fibrin D-dimer FEU [Mass/volume] in Platelet poor plasma	LOINC:48065-7
Fibrinogen [Mass/volume] in Platelet poor plasma by Coagulation assay	LOINC:3255-7
Platelets [#/volume] in Blood	LOINC:26515-7
von Willebrand factor (vWf) multimers [Presence] in Platelet poor plasma	LOINC:32217-2
C reactive protein [Mass/volume] in Serum, Plasma or Blood	TNX:9063
INR in Blood by Coagulation assay	LOINC:34714-6
Prothrombin time (PT) in Plasma or Blood	TNX:9033
Prothrombin time (PT)	LOINC:5902-2
Activated partial thromboplastin time (aPTT) in Plasma or Blood	TNX:9031
aPTT in Platelet poor plasma by Coagulation assay	LOINC:14979-9

ICD10CM: International Classification of Diseases, Tenth Revision, Clinical Modification.
LOINC: Logical Observation Identifier Name and Codes.
TNX: TrinetX curated.

Supplementary Table 3. Risk of Venous Thromboembolism in Patients With Raynaud's Syndrome Compared With Those Without Raynaud's Syndrome, Stratified by Calendar Period (2015–2019 vs. 2020–2024)

	Raynaud's syndrome group		Non-Raynaud's syndrome group			
	N	No. of event	N	No. of event	HR (95% CI)	P for proportional hazards assumption
**2015-2019**						
Venous Thromboembolism	46252	1139	46252	921	1.28 (1.18–1.40)	0.031
Deep Vein Thrombosis	46252	742	46252	599	1.29 (1.15–1.43)	0.164
Pulmonary embolism	46252	574	46252	489	1.22 (1.08–1.37)	0.284
**2020-2024**						
Venous Thromboembolism	59017	669	59017	596	1.25 (1.12–1.39)	0.804
Deep Vein Thrombosis	59017	444	59017	390	1.26 (1.10–1.45)	0.620
Pulmonary embolism	59017	330	59017	314	1.17 (1.001–1.36)	0.419

HR, Hazard Ratio; CI, Confidence interval.

Supplementary Table 4. Negative control outcome analysis in patients with and without Raynaud's syndrome

	Raynaud's syndrome group		Non-Raynaud's syndrome group			
	N	No. of event	N	No. of event	HR (95% CI)	P for proportional hazards assumption
Cataract	89570	4372	89570	4935	0.96 (0.92–1.00)	0.685
Burns and corrosions of external body surface, specified by site	87052	438	87052	469	1.01 (0.89–1.15)	0.847
Exposure to smoke, fire, and flames	92450	38	92450	48	0.87 (0.57–1.33)	0.397

HR, Hazard Ratio; CI, Confidence interval.
